# TLR3 signaling is either protective or pathogenic for the development of Theiler's virus-induced demyelinating disease depending on the time of viral infection

**DOI:** 10.1186/1742-2094-8-178

**Published:** 2011-12-21

**Authors:** Young-Hee Jin, Tomoki Kaneyama, Min Hyung Kang, Hyun Seok Kang, Chang-Sung Koh, Byung S Kim

**Affiliations:** 1 Department of Microbiology-Immunology, Northwestern University Medical School, Chicago, Illinois 60611, USA; 2 Department of Pathology, Graduate School of Medicine, Shinshu University, Matsumoto, Nagano 390-8621, Japan; 3 Biomedical Laboratory Sciences, Graduate School of Medicine, Shinshu University, Matsumoto, Nagano 390-8621, Japan

**Keywords:** TLR3, TMEV, demyelination, CNS, T cell responses

## Abstract

**Background:**

We have previously shown that toll-like receptor 3 (TLR3)-mediated signaling plays an important role in the induction of innate cytokine responses to Theiler's murine encephalomyelitis virus (TMEV) infection. In addition, cytokine levels produced after TMEV infection are significantly higher in the glial cells of susceptible SJL mice compared to those of resistant C57BL/6 mice. However, it is not known whether TLR3-mediated signaling plays a protective or pathogenic role in the development of demyelinating disease.

**Methods:**

SJL/J and B6;129S-*Tlr3^tm1Flv^*/J (TLR3KO-B6) mice, and TLR3KO-SJL mice that TLR3KO-B6 mice were backcrossed to SJL/J mice for 6 generations were infected with Theiler's murine encephalomyelitis virus (2 × 10^5 ^PFU) with or without treatment with 50 μg of poly IC. Cytokine production and immune responses in the CNS and periphery of infected mice were analyzed.

**Results:**

We investigated the role of TLR3-mediated signaling in the protection and pathogenesis of TMEV-induced demyelinating disease. TLR3KO-B6 mice did not develop demyelinating disease although they displayed elevated viral loads in the CNS. However, TLR3KO-SJL mice displayed increased viral loads and cellular infiltration in the CNS, accompanied by exacerbated development of demyelinating disease, compared to the normal littermate mice. Late, but not early, anti-viral CD4^+ ^and CD8^+ ^T cell responses in the CNS were compromised in TLR3KO-SJL mice. However, activation of TLR3 with poly IC prior to viral infection also exacerbated disease development, whereas such activation after viral infection restrained disease development. Activation of TLR3 signaling prior to viral infection hindered the induction of protective IFN-γ-producing CD4^+ ^and CD8^+ ^T cell populations. In contrast, activation of these signals after viral infection improved the induction of IFN-γ-producing CD4^+ ^and CD8^+ ^T cells. In addition, poly IC-pretreated mice displayed elevated PDL-1 and regulatory FoxP3^+ ^CD4^+ ^T cells in the CNS, while poly IC-post-treated mice expressed reduced levels of PDL-1 and FoxP3^+ ^CD4^+ ^T cells.

**Conclusions:**

These results suggest that TLR3-mediated signaling during viral infection protects against demyelinating disease by reducing the viral load and modulating immune responses. In contrast, premature activation of TLR3 signal transduction prior to viral infection leads to pathogenesis via over-activation of the pathogenic immune response.

## Background

Toll-like receptor 3 (TLR3) recognizes double stranded RNA (dsRNA), including poly IC and viral dsRNAs. TLR3 activation induces the production of a variety of cytokines, such as IL-1β, IL-6 and type I interferon (IFN) [1-4]. However, the role that TLR3 activation plays in the protection from or pathogenesis of virus-induced chronic disease is still unclear. It has been reported that a dominant-negative TLR3 allele is associated with the development of herpes simplex encephalitis, suggesting that TLR3 plays a protective role in herpes simplex virus infection [5]. In addition, TLR3 appears to play a protective role against infections with West Nile virus (WNV) [6], Coxsackievirus B4 [7], and mouse cytomegalovirus [8]. However, a detrimental role of TLR3 in the induction of acute pneumonia following influenza A virus infection has also been reported [9]. In addition, several studies have indicated that TLR3-mediated signals play either no role or a pathogenic role in viral diseases. For example, a recent study demonstrated that the absence of TLR3 did not alter viral pathogenesis after infection with single-stranded or double-stranded RNA viruses, such as lymphocytic choriomeningitis virus, vesicular stomatitis virus, and reovirus [10]. Furthermore, TLR3-deficient mice were more resistant to lethal WNV infection, although a TLR3-mediated signal was critical for the virus to penetrate into the brain where it caused neuropathogenesis [11].

Theiler's murine encephalomyelitis virus (TMEV) is a positive sense single-stranded RNA (ssRNA) virus of the *Picornaviridae *family [12]. TMEV establishes a persistent CNS infection in susceptible mouse strains that results in the development of demyelinating disease, which is considered a relevant viral model for human multiple sclerosis [13-15]. It has previously been shown that TLR3 recognizes the dsRNAs generated as TMEV replication intermediates, and TLR3 is essential for the production of TMEV-induced inflammatory cytokines, such as type I IFNs [16,17]. TLR3 is constitutively expressed in a variety of cells, including antigen presenting cells (dendritic cells and macrophages) as well as glial cells, including microglia and astrocytes [18]. In addition, the expression level of TLR3 is upregulated following TMEV infection and its expression levels are particularly high in cells from susceptible mice [19,20]. Furthermore, antigen presenting cells in the periphery and glial cells in the CNS are much more permissive to TMEV infection and support viral replication better than cells from resistant mice [21,22]. The differences appear to be, in part, due to the high intrinsic activation state of NF-κB in cells from susceptible mice [23]. TLR3-mediated signals activate multiple NF-κB pathways and upregulate the expression of other TLRs, such as TLR2, and following TMEV infection, these secondary TLRs contribute to the production of additional proinflammatory cytokines [17,24]. However, dsRNAs, including synthetic dsRNA poly IC, are recognized not only by TLR3 but also by MDA5 and PKR [16,24]. Therefore, the relative role of TLR3-mediated signaling in the development of TMEV-induced demyelinating disease remains to be determined.

In particular, the induction of strong type I IFN production, following infection with TMEV, is mediated by TLR3 and MDA5-mediated signals [16,17,24,25]. Our previous results showed that type I IFN was critical for the prevention of rapid fatal encephalitis, by controlling the viral load and the infiltration of inflammatory cells into the CNS [26]. However, type I IFN levels were significantly higher in susceptible SJL mice compared to resistant C57BL/6 mice [22]. Interestingly, type I IFNs play dichotomous roles in stimulating the immune responses, i.e., up- or down-regulating T cell responses, apparently depending on IFN concentration [21,27]. Furthermore, the time of type I IFN presence seems to be an important factor for the function of type I IFNs against viral infection [21]. Many recent studies utilized poly IC to activate TLR3 and/or MDA5-mediated signals in conjunction with viral infections and/or autoimmunity. For example, poly IC treatment of virus-infected mice resulted in a type I IFN-dependent reduction in viral loads and protection from virus-induced disease by enhancing the function of virus-specific T cells [28,29]. However, treatment with poly IC enhances the development of autoimmune diseases [30-32]. Therefore, it would be important to investigate the effects of different levels of type I IFNs that are activated via TLR3 in resistant and susceptible mice to determine its impact on the development of TMEV-induced demyelinating disease, which bears both viral and autoimmunity components.

To investigate the role of TLR3-mediated innate immune responses on the pathogenesis of TMEV-induced demyelinating disease, we utilized TLR3-deficient mice in both the resistant C57BL/6 (B6) and susceptible SJL/J backgrounds. In addition, we administered poly IC to activate TLR3-mediated signals prior to or after TMEV infection. Our results showed that TLR3-deficient susceptible SJL mice accelerated the development of demyelinating disease, whereas TLR3-deficient resistant B6 mice remained disease free. The virus-infected TLR3-deficient SJL mice displayed increased cellular infiltration and an elevated viral load in the CNS. Therefore, TLR3-mediated signals are important in protecting susceptible mice from the development of TMEV-induced demyelinating disease, although TLR3-mediated signals appear to play a minor role in resistant mice. However, treatment with poly IC prior to viral infection exacerbated disease development in susceptible mice, while treatment after viral infection somewhat ameliorated it. This observation suggests that either a premature activation or an over-activation of TLR3 signaling during early viral infection may lead to pathogenesis, perhaps through the development of a pathogenic immune response. Therefore, our current results strongly warrant caution on the use of TLR3-mediated immune interventions against chronic viral diseases and suggest careful consideration for these treatments in conjunction with the time of viral infection.

## Materials and methods

### Mice

SJL/J mice were purchased from the Charles River Laboratories (Charles River, MA) through the National Cancer Institute (Frederick, MD). B6; 129S-*Tlr3^tm1Flv^*/J mice (TLR3KO-B6) were purchased from Jackson Laboratories (Bar Harbor, ME). TLR3KO-B6 mice were backcrossed to SJL/J mice for 6 generations to obtain TLR3KO-SJL mice. The absence/presence of TLR3 in TLR3KO-SJL and the littermate mice (NLM) were typed based on the electrophoresis patterns of TLR3 and neomycin resistant genes. PCR products from tail genomic DNA of NLM and TLR3KO-SJL mice were determined using PCR-based genotyping analysis established by the Jackson Laboratory (Additional file 1, Figure S1). Experimental procedures that were approved by the Animal Care and Use Committee of Northwestern University in accordance with NIH animal care guidelines were used in this study.

### Virus

The BeAn and GDVII strains of TMEV were propagated in BHK-21 cells grown in DMEM medium supplemented with 7.5% donor calf serum. Viral titer was determined by plaque assay on BHK cell monolayers. The cells were incubated for 4-5 days in infection-medium (DMEM supplemented with 0.1% bovine serum albumin) with TMEV at 10 MOIs and the cell lysates were cleared by centrifugation. The cleared lysates yield 3-5 × 10^8 ^PFU and a pooled batch was used as a viral stock. If necessary the viral stock was diluted in DMEM before inoculation.

### Assessment of clinical signs

Approximately 30 μl of TMEV was injected into the right hemisphere of 5- to 7-week-old mice anesthetized with isofluorane. Resistant B6 and TLR3KO-B6 mice were infected with 1 × 10^6 ^PFU and susceptible SJL and TLR3KO-SJL mice were infected with 2 × 10^5 ^PFU TMEV. Clinical symptoms of disease were assessed weekly on the following grading scale: grade 0 = no clinical signs; grade 1 = mild waddling gait; grade 2 = moderate waddling gait and hindlimb paresis; grade 3 = severe hind limb paralysis; grade 4 = severe hind limb paralysis and loss of righting reflex; and grade 5 = death.

### Plaque assay

After cardiac perfusion with cold Hank's balanced salt solution (HBSS) (Mediatech), brain and spinal cords were removed. The tissues were homogenized in HBSS using a tissue homogenizer. A standard plaque assay was performed on BHK-21 cell monolayers [33]. Plaques in the BHK monolayer were visualized by staining with 0.1% crystal violet solution after fixing with methanol.

### Isolation of CNS-infiltrating lymphocytes

Mice were perfused through the left ventricle with 30 ml of sterile HBSS. Excised brains and spinal cords were forced through wire mesh and incubated at 37°C for 45 min in 250 μg/ml of collagenase type 4 (Worthington). CNS-infiltrating lymphocytes were then enriched at the bottom 1/3 of a continuous 100% Percoll (GE) gradient after centrifugation for 30 min at 27,000 × g.

### Flow cytometry

CNS-infiltrating lymphocytes were isolated and Fc receptors were blocked using 100 μl of 2.4G2 hybridoma (ATCC) supernatant by incubating at 4°C for 30 minutes. The indicated antibodies were subsequently used to stain various cell types. VP3_159-166_-loaded H-2K^s ^tetramer labeled with PE was used to assess levels of virus-specific CD8^+ ^T cells in the CNS of TMEV-infected mice. Cells were analyzed using a Becton Dickinson LSRII flow cytometer.

### Intracellular cytokine staining

Freshly isolated CNS-infiltrating mononuclear cells were cultured in 96-well round bottom plates in the presence of viral or control peptides and Golgi-Plug™ (BD) for 6 h at 37°C. Cells were then incubated in 100 μl of 2.4G2 hybridoma (ATCC) supernatant for 30 minutes at 4°C to block Fc receptors. Anti-CD8 (clone 53-6.7) antibody or anti-CD4 (clone L3T4) antibody was added, and cells were incubated for an additional 30 minutes at 4°C. After two washes, intracellular IFN-γ staining was performed according to the manufacturer's instructions (BD) using PE-labeled rat monoclonal anti-IFN-γ (XMG1.2) antibody. Cells were analyzed by flow cytometry.

### RT-PCR and real-time PCR

Total RNA was isolated by TRIzol reagent (Invitrogen) and reverse transcribed to cDNA using Moloney murine leukemia virus reverse transcriptase (Invitrogen). The cDNAs were amplified with specific primer sets using the SYBR Green Supermix (Bio-Rad) on an iCycler (Bio-Rad). The sense and antisense primer sequences used for cytokines are as follows: TMEV (VP1), (5'-TGACTAAGCAGGACTATGCCTTCC-3' and 5'-CAACGAGCCACATATGCGGATTAC-3'); IL-1β, (5'-TCATGGGATGATAACCTGCT-3' and 5'-CCCATACTTTAGGAA-GACACGGAT-3'); IFN-α, (5'-ACCTCCTCTGACCCAGGAAG -3' and 5'-GGCTCTCCAGA-CTTCTGCTC-3'); IFN-β, (5'-CCCTATGGAGATGACGGAGA-3' and 5'-CTGTCTGCTGG-TGGAGTTGA-3'); IFN-γ, (5'-ACTGGCAAAAGGATGGTGAC-3' and 5'-TGAGCTCATT-GAATGCTT GG-3'); IL-10, (5'-GCCAAGCCTTATCGGAAATGATCC-3' and 5'-AGACA-CCTTGGTCTTGGAGCTT-3'); TNF-α, (5'-CTGTGAAGGGAATGGGTGTT-3' and 5'-GGTCACTGTCCCAGCATCTT-3'); IL-6, (5'-AGTTGCCTTCTTGGGACTGA-3' and 5'-TCCACGATTTCCCAGAGAAC-3'); IL-17, (5'-GGGGATCCATGAGTCCAGGGAGAGC-3' and 5'-CCCTCGAGTTAGGCTGCCTGGCGGA-3'); CXCL10, (5'-AAGTGCTGCCGTC-ATTTTCT-3' and 5'-GTGGCAATGATCTCAACACG-3') and GAPDH, (5'-AACTTTGG-CATTGTGGAAGGGCTC-3' and 5'-TGCCTGCTTCACCACCTTCTTGAT-3'). GAPDH expression served as an internal reference for normalization. Real-time PCR was performed in triplicate.

### Statistical analyses

The statistical significance of the differences between experimental groups (two-tailed p value) was analyzed with the unpaired Student's t-test using the InStat Program (GraphPAD). Comparisons of the disease courses between 2 groups were also performed using the paired t-test. Values of *P *< 0.05 were considered to be significant.

## Results

### TLR3 deficiency in resistant B6 mice does not cause demyelinating disease by BeAn but results in elevated encephalitic death by the virulent GDVII strain of TMEV

**Figure 1:**
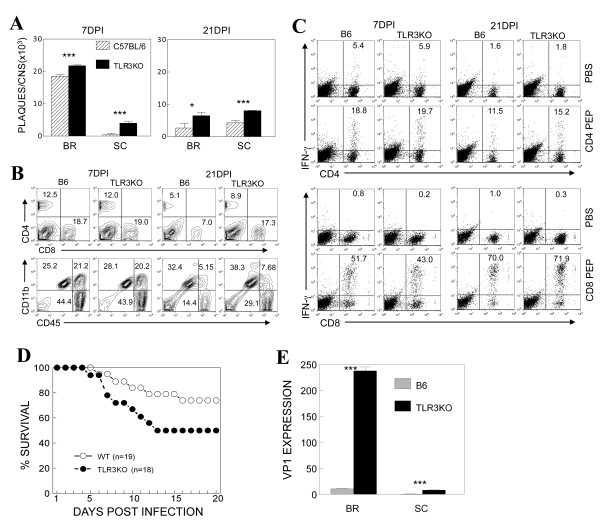
**Decreased TMEV clearance in the CNS of TLR3 KO mice**. (A) The level of infectious TMEV in the whole brain (BR) and spinal cord (SC) from wild type and TLR3 KO mice at 7 and 21 days postinfection (1 × 10^6 ^PFU) was determined by plaque assays in BHK cells. (*P *< 0.05 at all time points based on the paired Student's *t *test). Data represent values from a representative experiment from three independent experiments conducted with CNS pools of three mice per group. The values given are means ± standard deviations of triplicates. Statistically significant differences were indicated with asterisks (*, *P *< 0.05; ***, *P *< 0.001). (B) CNS-infiltrating mononuclear cells from TMEV-infected wild type and TLR3 KO mice at 7 and 21 dpi. Numbers in FACS plots represent percentages in the CNS. Data are representative of three experiments using three mice per group. (C) Levels of IFN-γ producing CD4^+ ^and CD8^+ ^cells in the CNS were determined by intracellular staining after stimulation for 6 hr with 2 μM CD4 or CD8 viral epitope peptides. Numbers in the FACS plots represent % of IFN-γ producing CD4^+ ^or CD8^+ ^cells from total infiltrating CD4^+ ^or CD8^+ ^cells. The data represent three separate experiments using three mice per group. (D) Survival rate of wild type and TLR3KO mice after intraperitoneal infection with GDVII was monitored for 20 days. (E) Viral persistence levels in the brain (BR) and spinal cord (SC) of infected mice at 7 dpi (DPI) were determined by quantitative PCR. Data are expressed by fold induction after normalization to GAPDH mRNA levels. The values given are means ± standard deviations of triplicates. Statistically significant differences were indicated with asterisks (***, *P *< 0.001)

It has previously been reported that TLR3 plays a critical role in TMEV-induced inflammatory cytokine and chemokine responses [16,17]. To examine the role of TLR3 in the development of TMEV-induced disease, we compared the development of clinical signs and viral loads in the CNS of control B6 and TLR3-deficient B6 (TLR3KO-B6) mice following infection with the BeAn strain of TMEV (1 × 10^6 ^PFU). Viral levels in the CNS of TLR3KO-B6 mice at 7 and 21 days post-infection (dpi) were significantly higher in the brain and spinal cord than the viral levels of the B6 control mice (Figure 1A). However, neither the B6 nor the TLR3KO-B6 mice developed detectable clinical signs of disease (data not shown). These results indicate that TLR3 signals are important in controlling TMEV loads in the CNS, although the increased viral levels did not lead to the development of demyelinating disease. Flow cytometric analysis of the CNS cells indicated that the level of mononuclear cells, including T cells and macrophages, that infiltrated the CNS of the TLR3KO-B6 mice were similar to those of the B6 mice at 7 dpi (Figure 1B). However, the levels of these cells in the CNS of TLR3KO-B6 mice were significantly higher than those of control B6 mice at 21 dpi. The elevated viral load in the TLR3KO mice may have caused a higher cellular infiltration into the CNS by activating higher levels of inflammatory cytokines and chemokines. To further determine the levels of virus-specific CD4^+ ^and CD8^+ ^T cells that infiltrated into the CNS, mononuclear cells were isolated from the CNS of TMEV-infected mice at 7 and 21 dpi, and these cells were stimulated with TMEV-specific viral epitope peptides. Subsequently, the ability of these T cells to produce IFN-γ was assessed by flow cytometry after intracellular cytokine staining (Figure 1C). The proportions of IFN-γ-producing, TMEV-specific CD4^+ ^T cells and CD8^+ ^T cells in the CNS were similar between TLR3KO-B6 and B6 mice.

As there were no differences in the development of TMEV BeAn-induced demyelinating disease between TLR3KO-B6 and B6 control mice, we further explored the potential differences in the susceptibility of these mice to a highly virulent GDVII strain of TMEV [34] that was administered via intraperitoneal injection (Figure 1D). After a low dose of viral infection (100 PFU), fewer than 26% of the control B6 mice developed fatal encephalitis, whereas greater than 50% of TLR3KO-B6 mice developed disease. In addition, virus-infected TLR3KO mice showed significantly higher levels of viral load in the CNS at 7 dpi compared to the infected B6 mice (Figure 1E), consistent with the differences noted in disease severity. These results indicate that TLR3-mediated signaling in resistant B6 mice plays an important role in controlling viral infection, particularly for highly virulent, encephalitic strains of TMEV.

### TLR3-deficient SJL (TLR3KO-SJL) mice are more susceptible to BeAn-induced demyelinating disease than SJL mice

**Figure 2:**
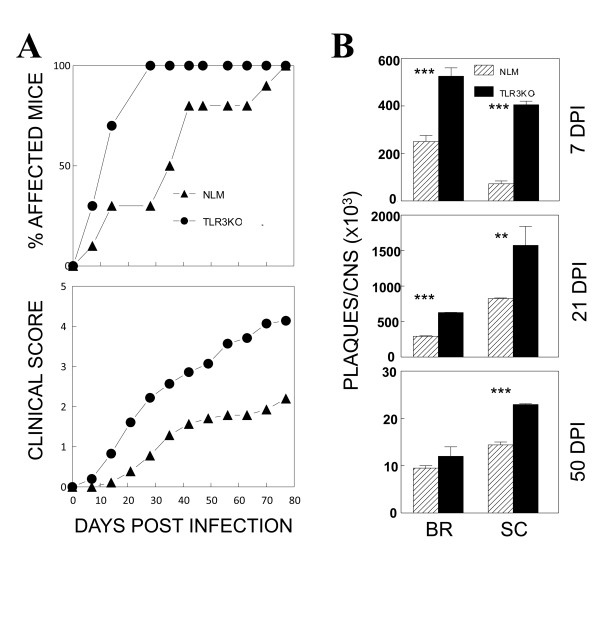
**The course of TMEV-induced demyelinating disease development and viral persistence levels in NLM and TLR3KO-SJL mice**. (A) Frequency and severity of demyelinating disease in NLM (n = 10) and TLR3KO-SJL (n = 10) were monitored for 80 days after TMEV infection. (B) Viral persistence levels in the brain (BR) and spinal cord (SC) of infected mice at 7, 21 and 50 dpi (DPI) were determined by plaque assay. Data represent values from a representative experiment from three independent experiments conducted with CNS pools of three mice per group. Statistically significant differences were indicated with asterisks (**, *P *< 0.01; ***, *P *< 0.001).

To examine whether TLR3 plays a more prominent role in TMEV-susceptible SJL mice, we infected TLR3KO-SJL mice and normal littermates (NLM) at the 6^th ^generation of backcrossing to SJL/J mice with a low dose (2 × 10^5 ^PFU) of TMEV BeAn. These virus-infected mice were then assessed for the progression of demyelinating disease for 80 dpi (Figure 2A). We chose the low dose of the virus to maximize the differences in disease development. Interestingly, TLR3KO-SJL mice showed exacerbated development of TMEV-induced demyelinating disease compared to the NLM. We further examined the viral loads in the CNS (brains and spinal cords) of both mouse groups at 7, 21 and 50 dpi using plaque assays (Figure 2B). The levels of infectious virus in the CNS of TLR3KO-SJL mice were significantly higher both in the brain and spinal cord compared to those for the control NLM mice. These results indicate that TLR3 signaling plays an important role in controlling viral load in the CNS and in preventing the development of TMEV-induced demyelinating disease following infection with a less virulent BeAn strain in mice of the susceptible SJL background, unlike mice of the resistant B6 background.

### TLR3KO-SJL mice display severe demyelination and inflammation in the CNS

**Figure 3:**
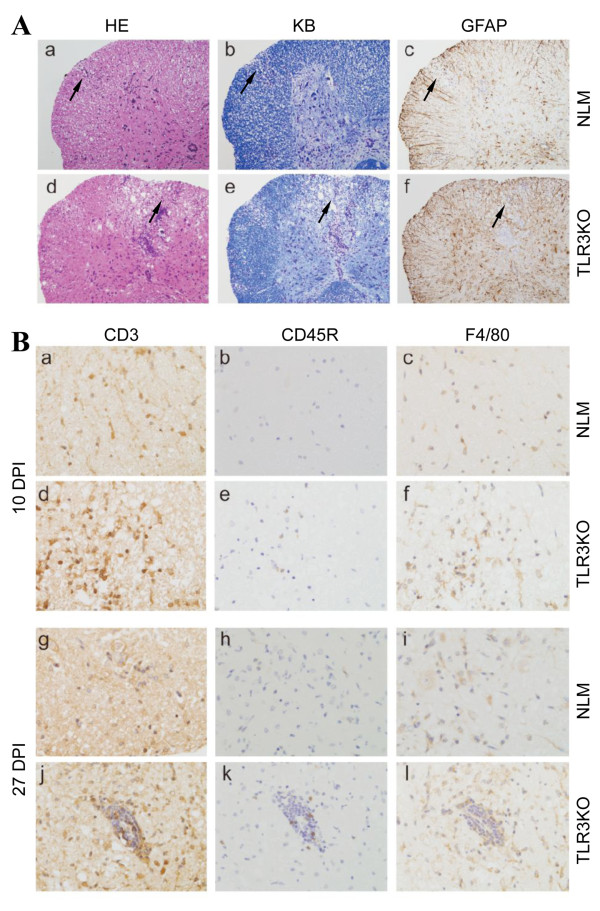
**Histopathology of TMEV-infected NLM and TLR3KO-SJL mice**. (A) Hematoxylin-eosin (HE) staining (a, d), Kluver-Barrera (KB) staining (b, e) and immunohistochemical staining for GFAP, astrocyte marker (c, f) of spinal cord from NLM or TLR3KO-SJL mice were done at 27 dpi (DPI). Original magnification, 100×. (B) Immunohistochemical staining for CD3 (a, d, g, and j), CD45R, a marker of B cell (b, e, h, and k), and F4/80, a marker of macrophage (c, f, i, and l) of the spinal cord from NLM or TLR3KO-SJL mice were done at days 10 and 27 post infection. Original magnification, 400×.

To compare levels of demyelination in the CNS of TMEV BeAn-infected TLR3KO-SJL and NLM SJL mice, histopathologic examinations were performed (Figure 3). First, Hematoxylin-eosin (HE) staining (Figure 3Aa and 3Ad), Kluver-Barrera's (KB) staining (Figure 3Ab and 3Ae) and immunohistochemical staining for GFAP (Figure 3Ac and 3Af) were conducted. In each experiment, mice from the NLM or TLR3KO groups were blindly selected, beforehand, for histological examination, and these mice were sacrificed at 27 dpi. The HE staining results showed that slight mononuclear cell infiltration (arrow) and mild demyelination were observed in the white matter of the spinal cord from NLM mice (Figure 3Aa and 3Ab). GFAP staining showed a lack of astrocytes in the demyelinated lesion (arrow) (Figure 3Ac). In contrast, markedly increased mononuclear cell infiltration (arrow) and extended demyelination (arrow) were observed in the white matter of the spinal cord from TLR3KO mice (Figure 3Ad and 3Ae). GFAP staining showed markedly increased number of activated astrocytes in the white matter of the spinal cords of these mice (Figure 3Af).

Next, we examined the spinal cords of TMEV-infected NLM and TLR3KO-SJL mice at days 10 and 27 post infection using immunohistochemical staining for CD3, a marker of T cells (Figure 3Ba, 3Bd, 3Bg, and 3Bj); CD45R, a marker of B cell (Figure 3Bb, 3Be, 3Bh, and 3Bk); and F4/80, a marker of macrophages (Figure 3Bc, 3Bf, 3Bi, and 3Bl). Increased T cell infiltration was observed in the white matter of the spinal cord from TLR3KO mice (Figure 3Bd and 3Bj) compared to NLM mice (Figure 3Ba and 3Bg), based on immunohistochemical staining for CD3. Few B cells were observed in the NLM (Figure 3Bb) and the TLR3KO mice at day 10 post-infection (Figure 3Be). On day 27 post-infection, B cell infiltration was increased in the white matter of the spinal cord from TLR3KO mice (Figure 3Bk) compared to NLM mice (Figure 3Bh). Similarly, macrophage infiltration was determined by staining for the F4/80 marker, and higher levels of macrophages were found in the white matter of the spinal cord from TLR3KO mice (Figure 3Bf and 3Bl) compared to NLM mice (Figure 3Bc and 3Bi) at 10 and 27 dpi.

### Levels of cellular infiltration, viral load and type I IFN production are elevated in the CNS of TLR3-deficient mice

**Figure 4:**
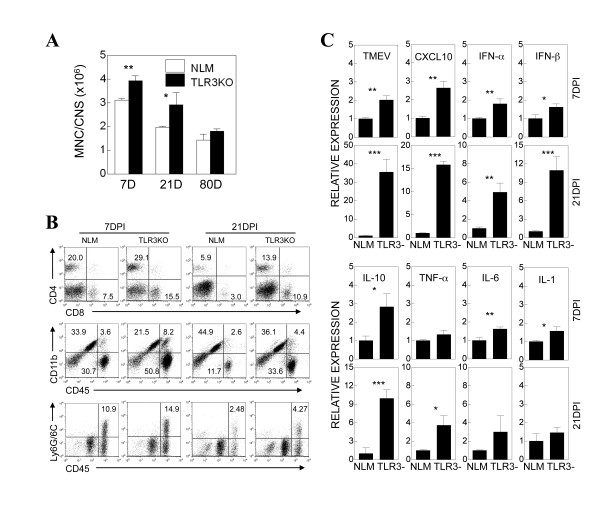
**CNS-infiltrating mononuclear cells from TMEV-infected NLM and TLR3KO-SJL mice**. (A) Overall numbers of CNS-infiltrating mononuclear cells in the CNS of TMEV-infected NLM and TLR3KO-SJL at 7, 21, and 80 DPI were shown. The values given are means ± standard deviations of 2-3 independent experiments derived from pooled cells of 3-4 mice per group. (B) Levels of T cells (CD4^+ ^and CD8^+^), macrophages (CD11b^+ ^CD45^high^) and granulocytes (Ly6G/6C^+ ^and CD45^+^) were assessed using flow cytometry. Numbers in the FACS plots represent percentages in the CNS of TMEV-infected NLM and TLR3KO-SJL at 7 and 21 dpi. (C) Cytokine mRNA expression levels in the CNS of NLM and TLR3KO-SJL at 7 and 21 dpi were analyzed by quantitative PCR. Data are expressed by fold induction after normalization to the GAPDH mRNA levels. The values given are means ± standard deviations of triplicates. Statistically significant differences were indicated with asterisks (*, *P *< 0.05; **, *P *< 0.01; ***, *P *< 0.001). A representative result of three separate experiments using three mice per group was shown here.

To determine the levels of CNS-infiltrating mononuclear cells, we compared the mononuclear cells that accumulated in the CNS of NLM and TLR3K-SJL mice. The numbers of CNS-infiltrating mononuclear cells were elevated throughout the course of viral infection (days 7, 21 and 80) in TLR3KO-SJL mice compared to NLM (Figure 4A). Flow cytometric analysis of the CNS infiltrating mononuclear cells indicated that the proportions of both the CD4^+ ^and CD8^+ ^T cells in TLR3KO-SJL mice were significantly higher than those of the NLM group at 7 and 21 dpi (Figure 4B). The proportions of macrophages (CD11b^+^CD45^high^) and neutrophils (Ly6G/6C^+^) were also higher in TLR3KO-SJL mice compared to NLM.

To understand the underlying mechanisms of exacerbated susceptibility to TMEV-induced demyelinating disease in TLR3KO-SJL mice, we compared the expression levels of TMEV RNA and cytokine genes in the CNS of virus-infected NLM and TLR3KO-SJL mice at 7 and 21 dpi (Figure 4C). The viral message level was significantly elevated at both time points in TLR3KO-SJJL mice, consistent with the higher replicating virus levels determined using plaque assays (Figure 2B). The expression of various inflammatory cytokine genes, such as type I IFNs, IL-10, TNF-α, IL-6, and IL-1 was similarly elevated in the CNS of TLR3KO-SJL mice. It was interesting to note that the expression of CXCL-10, associated with T cell infiltration to the CNS, was also highly elevated in TLR3KO-SJL mice. Because TLR3 is known to play an important role in activating the expression of these cytokine genes [16], an increased viral load in the absence of TLR3 signaling may be sufficient to overcome the TLR3 deficiency via other receptors, such as MDA5, leading to elevated cytokine gene expression. Consequently, higher viral loads accompanied by more proinflammatory cytokines may result in elevated cellular infiltration and exacerbated development of demyelinating disease in TLR3KO-SJL mice.

### Late, but not early, anti-viral T cell responses are compromised in TLR3KO-SJL mice

**Figure 5:**
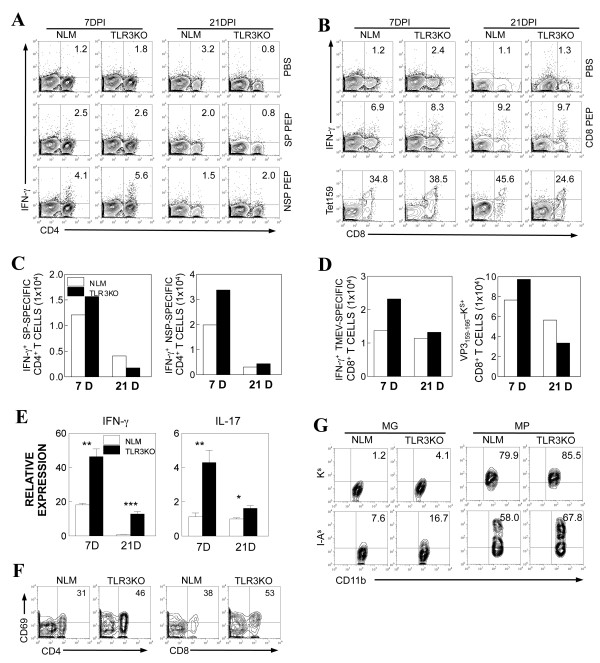
**CD4^+ ^and CD8^+ ^T cell responses to viral epitopes in TMEV-infected NLM and TLR3KO-SJL mice**. (A) Proportions of IFN-γ-producing CD4^+ ^cells in the CNS were determined by intracellular staining after stimulation with 2 μM of SJL CD4 capsid (SJL SP) or SJL CD4 noncapsid (SJL NSP) epitope mixtures. Numbers in the FACS plots represent % of IFN-γ producing CD4^+ ^cells from total infiltrating CD4^+ ^cells. (C) IFN-γ-producing CD4^+ ^cell numbers in the CNS at 7 and 21 dpi were shown after stimulation with SJL CD4 capsid (SJL SP) (left panel) or SJL CD4 noncapsid (SJL NSP) (right panel) epitope mixtures. (B) Proportions of IFN-γ-producing CD8^+ ^cells in the CNS were determined by intracellular staining after stimulation with 2 μM of SJL CD8 epitope mixture. Numbers in the FACS plots represent % of IFN-γ producing CD8^+ ^cells from total infiltrating CD8^+ ^cells. Numbers in the bottom panel represent % of H-2K^s^-VP3_159-166 _tetramer-positive CD8^+ ^cells from total infiltrating CD8^+ ^cells without further stimulation. (D) IFN-γ-producing CD8^+ ^cell numbers after stimulation with SJL CD8 epitopes (left panel) and total numbers of H-2K^s^-VP3_159-166 _tetramer-reactive CD8^+ ^cells in the CNS at 7 and 21 dpi were shown (right panel). (E) The relative expression levels of IFN-γ vs. IL-17 mRNAs in the CNS of virus-infected TLR3KO-SJL and NLM were assessed by real-time PCR. Data are expressed by fold induction after normalization to the GAPDH mRNA levels. The values given are means ± standard deviations of triplicates. Statistically significant differences were indicated with asterisks (*, *P *< 0.05; **, *P *< 0.01; ***, *P *< 0.001). (F) Expression of CD69, activation marker of CD4 and CD8 from TMEV-infected NLM and TLR3KO-SJL mice at 7 DPI was analyzed by FACS. Numbers in cytometric plots represent % of positive CD4^+ ^or CD8^+ ^T cells out of the total CD4^+ ^or CD8^+ ^T cells, respectively. (G) Expression levels of MHC class I (H-2K^s^) and II (I-A^s^) molecules on microglia (MG) and macrophages (MP) of TMEV-infected NLM and TLR3KO-SJL mice were analyzed at 7 dpi by flow cytometry. Numbers in cytometric plots represent % of positive MG or MP out of the total MG or MP, respectively.

To determine the levels of virus-specific T cell responses in the CNS, mononuclear cells isolated from the CNS of TMEV-infected NLM and TLR3KO-SJL mice at 7 and 21 dpi were stimulated with viral epitope peptides and assayed for the production of IFN-γ (Figure 5A-D). The proportion of TMEV-specific IFN-γ-producing CD4^+ ^T cells and CD8^+ ^T cells in the CNS of TLR3KO-SJL mice were consistently similar or higher than those of the NLM mice at 7 dpi. The proportion of H-2K^s^-VP3_159-166_-tetramer reactive CD8^+ ^T cells in the CNS of TLR3KO mice was also similar to that of the NLM mice at the early stage (7 dpi) of infection, indicating the similarities in the function of virus-specific CD8^+ ^T cells in both mouse groups. However, the overall numbers of virus-specific CD4^+ ^and CD8^+ ^T cells in the CNS were higher due to the increased cellular infiltration to the CNS in TLR3KO-SJL mice. The proportion and number of anti-viral CD4^+ ^T cells became similar or lower in the mice at 21 dpi. Similarly, VP3_159-166_-tetramer reactive CD8^+ ^T cells were lower at 21 dpi in the TLR3KO-SJL mice, although IFN-γ-producing CD8^+ ^T cells remained similar. It has previously been shown that Th17 cells are preferentially developed following TMEV infection and IL-17 promotes the pathogenesis of chronic demyelinating disease [35]. To further determine the levels of IL-17-producing T cells relative to IFN-γ-producing cells in the virus-infected mice, the overall levels of IFN-γ and IL-17 messages expressed in the CNS were assessed using real-time PCR (Figure 5E). The results confirmed the higher level of IFN-γ-producing cells observed by flow cytometry in virus-infected TLR3KO-SJL mice. In addition, the level of IL-17-producing T cells was similarly higher in TLR3KO mice compared to the control littermates. These results suggest that antiviral T cell responses are not drastically altered but rather, are elevated in TMEV-infected SJL mice in the absence of TLR3 signals.

To further examine the status of T cell activation in TLR3KO-SJL mice during early viral infection, the expression of the CD69 activation marker on T cells and MHC molecules on microglia and macrophages in the CNS of virus-infected mice were analyzed at 7 dpi by flow cytometry (Figure 5F and 5G). Levels of CD69 expression on CD4^+ ^and CD8^+ ^T cells were higher in the TLR3KO-SJL mice compared to NLM mice, consistent with the higher proportions of virus-specific T cells (Figure 5A and 5B). The expression levels of both MHC class I (H-2K^s^) and II (I-A^s^) molecules were also higher on microglia (MG) and macrophages (MP) from TMEV-infected TLR3KO mice (Figure 5G). These results suggest that early efficient T cell activation, in the absence of TLR3 signaling, may be due to the elevated expression of MHC molecules on antigen presenting cells.

### Treatment of SJL mice with poly IC prior to viral infection, not after infection, exacerbates disease development, accompanied with elevated cellular infiltration to the CNS

**Figure 6:**
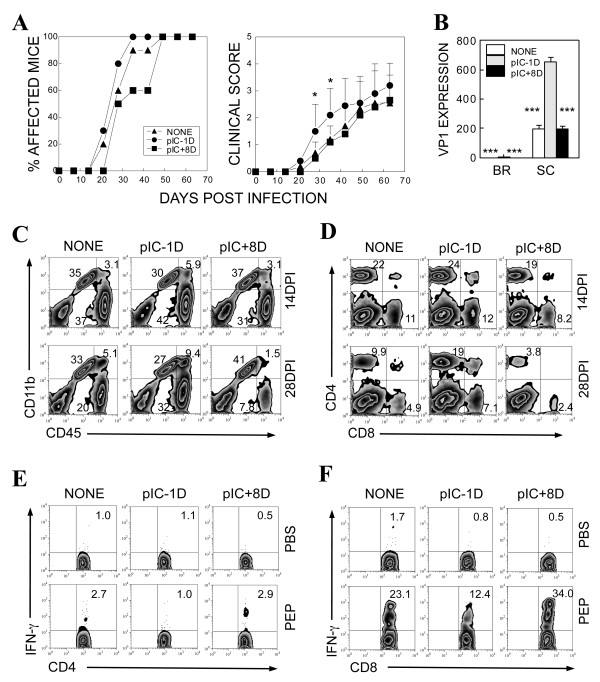
**Administration of poly IC at 1 day prior to or 8 days post TMEV infection into SJL mice**. Mice were intraperitoneally injected with either PBS or 50 μg of poly IC (Sigma) in 100 μl at day -1 or +8 relative to viral infection. (A) Frequency and severity of demyelinating disease in SJL mice after ip injection with poly IC at 1 day prior to (n = 10) or 8 days post (n = 10) TMEV infection were monitored for 63 days after TMEV infection. Statistically significant differences in Student's t-test were indicated with asterisks (*, *P *< 0.05). Paired t-tests (two-tailed) between 20-63 dpi indicated that the difference between untreated and pretreated groups was very significant (*P *< 0.0026) and the difference between untreated and post-treated groups was significant (*P *< 0.0189). (B) Viral persistence levels in the brain (BR) and spinal cord (SC) of infected mice at 14 dpi were determined using quantitative PCR. Data are expressed by fold induction after normalization to the GAPDH mRNA levels. The values given are means ± standard deviations of triplicates. Statistically significant differences were indicated with asterisks (*, *P *< 0.05; **, *P *< 0.01; ***, *P *< 0.001). (C and D) CNS-infiltrating mononuclear cells from SJL mice with treatment of poly IC at -1 day or +8 days post TMEV infection were shown. Numbers in FACS plots represent percentages in the total CNS-infiltrating cells. Data are representative of three experiments using three mice per group. (E) Levels of IFN-γ producing CD4^+ ^cells in the CNS were determined by intracellular staining after stimulation for 6 hours with 2 μM CD4 epitope peptides at 28 dpi. (F) Levels of IFN-γ producing CD8^+ ^cells in the CNS were determined by intracellular staining after stimulation for 6 hours with 2 μM CD8 viral epitope peptides at 28 dpi. Numbers in the FACS plots represent percentages in total CD4 or CD8 cells. Data are representative of three experiments using three mice per group.

To activate TLR3 signaling, susceptible SJL mice were intraperitoneally treated with poly IC, the representative TLR3 ligand, at 1 day prior to or 8 days post TMEV-infection. The progression of TMEV-induced demyelinating disease was assessed over 63 dpi. Mice treated with poly IC at 1 day prior to viral infection displayed an exacerbated development of disease, whereas mice treated with poly IC at 8 dpi resulted in a slower onset of the disease compared to virus-infected SJL mice without poly IC administration (Figure 6A). Viral message levels in the brain and spinal cord of mice pretreated with poly IC were significantly higher than those of the mice that were either untreated or treated with poly IC at 8 dpi (Figure 6B). These results indicate that the activation of TLR3 prior to viral infection leads to an increased viral load in the CNS and accelerated pathogenesis of demyelinating disease; however, such activation after viral infection does not alter the development of disease.

To further understand the immunological mechanisms of the acceleration of TMEV-induced demyelinating disease in poly IC-pretreated SJL mice, we first compared the levels of mononuclear cells accumulated in the CNS of mice at 14 and 28 dpi (Figure 6C and 6D). Flow cytometric analysis showed that the proportion of macrophages (CD11b^+^CD45^high^) in the CNS of poly IC pretreatment mice was elevated, whereas the proportion in the CNS of poly IC post-treated mice remained the same as that of virus-infected mice without poly IC treatment (Figure 6C). It is interesting to note that poly IC-pretreated mice maintained the elevated macrophage level at 28 dpi, while in poly IC-post-treated mice the level decreased. Similarly, the proportion of CD4^+ ^and CD8^+ ^cells was higher in the CNS of the poly IC pre-treatment mice and remained higher at 28 dpi compared to the untreated virus-infected mice. However, the proportion of these T cells in the CNS of poly IC-post-treated mice was lower (Figure 6D), and these decreases appear to reflect decreased levels of viral message in the CNS (Figure 6B).

To further assess the relative levels of virus-specific T cell responses in the CNS of these poly IC treated mice, mononuclear cells isolated from the CNS of infected mice at 14 and 28 dpi were stimulated with viral epitope peptides to determine the ability of these cells to produce IFN-γ. The flow cytometry profiles of the mononuclear cells at day 28 post-infection are shown in Figure 6 (panels E and F). The proportions of both IFN-γ-producing virus-specific CD4^+ ^and CD8^+ ^T cells in the CNS of poly IC pre-treated mice were markedly decreased at both time points (results at day 14 post-infection not shown) compared to those of the control mice without poly IC-treatment. In contrast, the proportion of CD4^+ ^and CD8^+ ^T cells in the poly IC-post-treated mice were increased particularly around the onset of disease development (28 dpi). These results strongly suggest that activation of TLR3 signaling prior to viral infection hinders the induction of protective IFN-γ-producing CD4^+ ^as well as CD8^+ ^T cell populations. In contrast, activation of these signals after viral infection appears to improve the induction of IFN-γ-producing CD4^+ ^as well as CD8^+ ^T cells.

### Expression of antigen presentation-associated molecules is elevated in CNS CD11b^+ ^cells in poly IC-pretreated mice but reduced in post-treated mice

**Figure 7:**
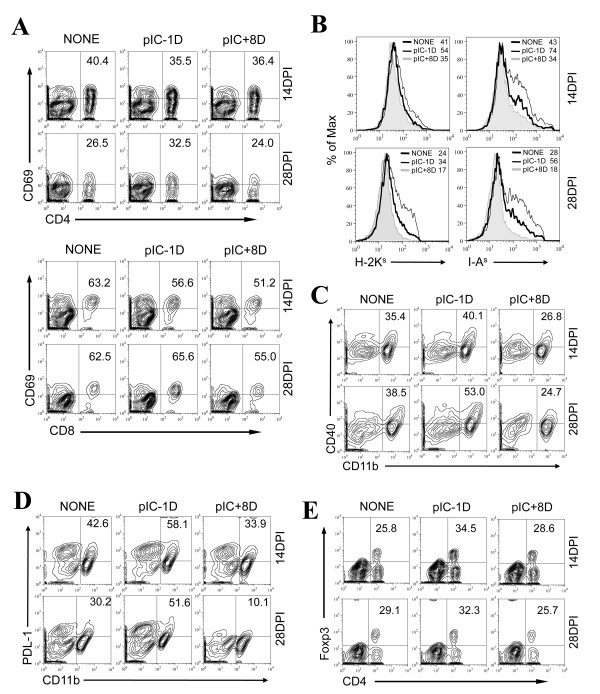
**Expressions of CD69, MHC molecules, CD40, PDL-1, and Foxp3 on CNS cells**. Expressions of CD69 and FoxP3 molecules on T cells, and MHC, CD40 and PDL-1 molecules on CD11b^+ ^cells in the CNS of mice were determined at 14 and 28 dpi by flow cytometry. (A) Expression of the CD69 activation marker on CD4^+ ^and CD8^+ ^T cells. (B) Expression of MHC class I (H-2K^s^) and II (I-A^s^) on CD11b^+ ^cells (microglia and macrophages). Numbers in the FACS plots represent Mean Fluorescence Intensity. Expressions of CD40 (C) and PDL-1 (D) on CNS CD11b^+ ^cells are shown. Numbers in the FACS plots represent percentages in total CD11b^+ ^cells. (E) Expression of Foxp3 on CD4^+ ^cells. Numbers in FACS plots represent percentages of total CD4^+ ^cells. Data are representative of three experiments using three mice per group.

To further determine whether the decrease of IFN-γ-producing T cells in poly IC-pretreated mice (Figure 6E and 6F) reflects the inability of antigen presenting cells to stimulate T cell responses in the CNS of poly IC pre- or post-treated SJL mice, expression levels of CD69, an activation marker of CD4^+ ^and CD8^+ ^cells, were compared at 14 and 28 dpi (Figure 7A). Overall, the expression levels of CD69 on CD4^+ ^and CD8^+ ^cells were similar among the untreated control and the poly IC pre- and post-treated mice at both 14 and 28 dpi, although the expression of CD69 on CD8^+ ^T cells of poly IC-post-treated mice was somewhat lower. These data suggest that the decrease in IFN-γ-producing T cell responses in poly IC-pretreated mice does not reflect the status of T cell activation in the CNS.

To verify the status of antigen presenting cells in the CNS, we examined the expression levels of MHC classes I and II, and CD40 molecules, which are associated with T cell activation, on the major antigen-presenting CD11b^+ ^cells, including microglia and macrophages (Figure 7B and 7C). The expression levels of MHC class I (H-2K^s^) and II (I-A^s^) molecules on CD11b^+ ^cells in the poly IC pretreated mice were markedly increased, whereas the levels in the poly IC post-treated mice were decreased compared to those in the untreated control virus-infected mice. These data suggested that the poor IFN-γ-producing T cell responses were not due to a deficiency in the expression of molecules associated with antigen presentation. It was also interesting to note that the levels of both T cell activation and expression of MHC and CD40 molecules appeared to correlate with viral load in the CNS.

To further explore possible mechanisms underlying the poor IFN-γ-producing T cell responses in poly IC-pretreated mice, we assessed the expression levels of PDL-1, an inhibitory molecule for both CD4^+ ^and CD8^+ ^T cell responses [36], on CD11b^+ ^cells in the CNS (Figure 7D). We chose PDL-1 as a candidate inhibitory molecule because this molecule is known to play a critical role in anti-viral T cell functions in virus-infected hosts, and the expression of PDL-1 is inducible by activation of TLR3 with poly IC treatment [36,37]. The expression levels of PDL-1 on CD11b^+ ^cells were drastically increased in the poly IC pretreated mice at both 14 and 28 dpi, whereas the expression was markedly decreased in post-treated mice compared to those of TMEV-infected control mice without poly IC treatment. These results strongly suggest that the compromise in the immune response of mice treated with poly IC prior to virus infection was due in part to the over-expression of the inhibitory PDL-1 molecule on antigen presenting cells rather than deficiencies in the activation of T cells.

It is possible that the poor immune responses in the poly IC-pretreated mice may also have been associated with the induction of a higher level of regulatory FoxP3^+ ^CD4^+ ^T cells (Treg), which are known to inhibit the function of anti-viral T cell responses [38-42]. To examine this possibility, levels of Foxp3 expressing CD4^+ ^T cells in the CNS of control mice and mice treated with poly IC were assessed at 14 and 28 dpi (Figure 7E). The level of Treg cells in the CNS of poly IC-pretreatment mice was significantly higher, particularly at the preclinical stage (14 dpi) compared to that of untreated control mice. In contrast, the Treg levels in mice treated with poly IC following viral infection were similar to the untreated control mice. These results suggest that an elevated induction of FoxP3^+ ^Treg cells may also partially contribute to the low T cell response in poly IC-pretreated mice.

## Discussion

We have previously demonstrated that cells infected with TMEV stimulate the innate inflammatory response mainly via TLR3-mediated signaling [16,17]. However, the role of TMEV-induced TLR3 signaling in protection from and/or pathogenesis of demyelinating disease remains unknown. In this study, we examined the potential role of TLR3 in the progression of TMEV-induced demyelinating disease by utilizing TLR3 KO mice and administering TLR3 ligand. Our results demonstrate that TLR3-mediated signals do not play a major role in the protection of mice in the resistant C57BL/6 background against BeAn, a less virulent strain of TMEV. However, TLR3 stimulation plays a protective role in infection with GDVII, a neurovirulent TMEV strain (Figure 1). These results are consistent with previous studies demonstrating that the absence of TLR3 in B6 mice does not alter the adaptive immune response or viral pathogenesis of chronic viral infections [10]. In contrast, it has also been reported that the presence of TLR3 provides protection from acute viral infections with West Nile virus [6] and Coxsackievirus B4 [7]. Therefore, it appears that TLR3 may provide some protection against acute or virulent viral infections but not against non-virulent viral infections.

In contrast to resistant C57BL/6 mice, SJL mice are susceptible to persistent chronic infection in the CNS with the less virulent BeAn strain of TMEV, and the majority of infected mice develop demyelinating disease starting from 20-35 dpi [15]. Our current results indicate that the presence of TLR3-mediated signals provides protection from the development of TMEV-induced demyelinating disease in susceptible SJL mice, as TLR3-deficient mice with the SJL background genes showed elevated viral loads in the CNS and exacerbated disease development (Figures 2 and 3). Therefore, TLR3-mediated protection may play an important role in the susceptible host that only mounts a marginal protective response against chronic viral infections. While the early adaptive immune response to viral infections was not altered in the absence of TLR3-mediated signals (Figure 5), consistent with a previous report [10], cellular infiltration into the CNS was markedly elevated (Figure 4A and 4B), resulting in exacerbation of TMEV-induced immune-mediated demyelinating disease (Figure 2A). The increased cellular infiltration may be due to high viral loads in the absence of TLR3 signals (Figure 2B), which leads to high levels of proinflammatory cytokine production in the CNS, thus facilitating cellular infiltration (Figure 4C). However, the elevated cytokine production in the CNS of virus-infected TLR3KO mice was unexpected, as TLR3 is essential for the production of cytokines, such as type I IFNs and IL-6, in TMEV infected glial cells [16,17]. Therefore, these results strongly suggest that high viral loads in the CNS led to the utilization of an alternative innate immunity pathway, such as MDA5 and/or PKR, which stimulate proinflammatory cytokine production, as previously described [4,24,43,44]. Since cells from TLR3KO mice can also produce cytokines upon stimulation with poly IC, these alternative signal-triggering molecules appear to be operational in these mice (not shown). Nevertheless, TLR3-mediated signals appear to provide a protective function, particularly in hosts susceptible to virus-induced disease.

It is interesting to note that there is a disconnect between the levels of type I IFNs and control of TMEV infection, hence TRLR3KO mice display higher levels of type I IFNs yet more susceptible to TMEV infection (Figures 2 and 4). These results are inconsistent with the previous studies with IFNIR-KO mice, which displayed fatal encephalitis upon TMEV infection [26,45]. Therefore, the presence of a certain level of type I IFN signaling during early TMEV infection appears to be necessary for survival of the animals. The high level of type I IFN production in TLR3KO mice is likely activated via primarily MDA5 signaling by a high viral load, because an MDA5-mediated signal is the major activator for type I IFN production in mice following infection with TMEV [24,46]. However, high levels of type I IFNs may not be necessarily helpful in controlling viral infection. In fact, both IFN-α and IFN-β levels were significantly higher in mice pretreated with poly IC compared to either untreated or treated at 8 dpi (Additional file 2, Figure S2). Furthermore, our previous results indicated that susceptible SJL mice produce higher levels of type I IFNs compared to resistant B6 mice and a high level of IFNs exacerbates viral infection by inhibiting induction of protective immune responses [21]. Therefore, the exceeding levels of type I IFNs appear to play a detrimental role in the protection from virus-induced chronic demyelinating disease.

Interestingly, premature activation of TLR3 via administration of poly IC prior to viral infection promoted disease progression. In contrast, additional TLR3 signals by poly IC after viral infection yielded a clinical improvement and less pathogenic immune responses in the CNS (Figure 6). These results suggest that TLR3 signaling provides differential protection against viral infection, depending on the time of the signals with respect to viral infection. It was previously shown that the presence of external poly IC mainly stimulates TLR3-mediated signals for the production of various proinflammatory cytokines in many different cell types, including macrophages, microglia, and astrocytes [3,16,19]. Poly IC, a TLR3 ligand, has previously been used to protect the host from acute viral infections. Administration of poly IC between < 72 hours prior to infection and < 24 post infection with foot and mouth disease virus protected mice from death [47]. Similarly, poly IC treatment at 1 day prior to infection through 4 hours post virus challenge effectively prolonged the survival of mice from herpes simplex virus 2 challenges [28]. Therefore, the efficacy of TLR3-mediated protection from acute viral infection appears to be limited to a narrow time window. Furthermore, such poly IC treatment prior to viral infection may exacerbate the development of chronic virus-infection induced immune-mediated diseases, such as TMEV-induced demyelinating disease (Figure 6). Interestingly, it has recently been shown that poly IC treatment enhances autoimmune disease in a retinal autoimmunity model [32]. Therefore, it is conceivable that the exacerbation of virus-induced disease by pretreatment with poly IC may not be limited to the development of chronic viral infection-induced immune mediated disease.

In contrast to the treatment with poly IC prior to viral infection, poly IC administration at 8 days after TMEV infection ameliorated disease development (Figure 6A). Recently, it has been shown that poly IC treatment of mice at 4 and 8 days after infection with Friend retrovirus reduces viral loads and promotes protection from the development of chronic viral infection-induced leukemia over a period of several weeks [29]. Therefore, TLR3-mediated signaling during chronic viral infection, particularly infections leading to immune-mediated diseases, appears to be protective, whereas premature activation of the signals prior to and/or at the time of viral infection may exacerbate the pathogenesis.

Our further analyses of the immune response in poly IC treated mice showed marked reductions in protective, virus-specific IFN-γ-producing CD4^+ ^and CD8^+ ^T cell responses in poly IC pretreated mice, in contrast to increases in poly IC post-treated mice (Figure 6E and 6F). Furthermore, poly IC-pretreated mice displayed elevated expression of a T cell inhibitor, PDL-1, and an increased generation of regulatory FoxP3^+ ^CD4^+ ^T cells in the CNS, while poly IC-post-treated mice expressed reduced levels of PDL-1 and FoxP3^+ ^CD4^+ ^T cells (Figure 7). The engagement of PD-1/2 or CD80 with PDL-1 exerts a powerful inhibitory function for CD4^+ ^as well as CD8^+ ^T cells in many virus systems (13). In addition, poly IC treatment is also known to upregulate PDL-1 expression [36,37]. Furthermore, it is interesting to note that poly IC-pretreated mice uniquely showed an increased level of FoxP3^+ ^regulatory CD4^+ ^T cells in the CNS of virus-infected mice. Although the underlying mechanisms for the increase are unknown, elevated levels of cytokines in the CNS of mice with high viral loads favoring the generation of FoxP3^+ ^CD4^+ ^T cells may contribute to the increase of the regulatory T cells. Nevertheless, FoxP3^+ ^CD4^+ ^T cells generated in virus-infected hosts, including TMEV-infected mice, inhibit virus-specific CD4^+ ^as well as CD8^+ ^T cell function [42,48]. Therefore, these results strongly suggest that the activation of TLR3 signaling prior to virus infection may induce premature stimulation of regulatory immune mechanisms, hindering anti-viral immune cell function and leading to viral persistence. On the other hand, further activation of TLR3 signaling after viral infection appears to enhance anti-viral T cell function by reducing the expression of inhibitory PDL-1 and preventing the generation of regulatory T cells. These observations are particularly important, as the results imply that TLR-mediated stimulation of innate immunity as an intervention strategy for the treatment of viral infections could exacerbate the development of chronic immune-mediated disease. Therefore, the timing of innate immunity stimulation should be carefully considered.

## Conclusions

We reported previously that TLR3-mediated signaling is important in the induction of innate cytokine responses to TMEV infection. In this study, we investigated the role of TLR3-mediated signaling in the development of TMEV-induced demyelinating disease. TLR3KO mice in the susceptible SJL background displayed increased cellular infiltration and viral loads in the CNS, accompanied by exacerbated development of demyelinating disease. Activation of TLR3 with poly IC prior to viral infection also exacerbated disease development, whereas such activation after viral infection slowed disease development. An increased viral load in the absence of TLR3 signaling led to elevated cytokine production, cellular infiltration, and exacerbated development of demyelinating disease. Activation of TLR3 signaling prior to viral infection hindered the induction of protective IFN-γ-producing CD4^+ ^and CD8^+ ^T cell populations, but elevated PDL-1 expression and regulatory CD4^+ ^T cell generation in the CNS. These results suggest that TLR3-mediated signaling during viral infection protects against demyelinating disease by reducing the viral load. In contrast, premature activation of TLR3 signal transduction prior to viral infection may induce premature stimulation of regulatory immune mechanisms, hindering anti-viral immune cell function and promoting viral persistence.

## Abbreviations

TLR3**: **toll-like receptor-3; MS**: **multiple sclerosis; CNS: central nervous system; dpi: days post-infection; PFU: plaque forming units; RT-PCR: reverse transcriptase-polymerase chain reaction; TMEV: Theiler's murine encephalomyelitis virus.

## Competing interests

The authors declare that they have no competing interests.

## Authors' contributions

YHJ and MHK investigated disease development. YHJ performed immunological studies and contributed to writing the manuscript. HSK generated peptide-loaded tetramers. TK and CSK performed histological studies and contributed to analysis. CSK and BSK analyzed the data and wrote the manuscript. All authors have seen and approved the final version of the manuscript.

## Supplementary Material

Additional file 1 **Genotyping of the presence of TLR3**. The presence/absence of TLR3 in TLR3KO-SJL and the littermate mice (NLM) were typed based on the electrophoresis patterns of TLR3 and neomycin resistant genes. PCR products from tail genomic DNA of NLM and TLR£KO-SJL mice were determined using PCR-based genotyping analysis, established by the Jackson Laboratory. TLR3 primers (5'-ACT CCT TTG GGG GAC TTT TG-3 and 5'-CAG GTT CGT GCA GAA GAC AA-3') and Neo generic primers (5'-CTT GGG TGG AGA GGC TAT TC-3' and 5'-AGG TGA GAT GAC AGG AGA TC-3') were used for PCR. Click here for file

Additional file 2 **Levels of type I IFNs in poly IC-treated mice**. Type I interferon (IFN-α and IFN-β) levels in the CNS of TMEV-infected SJL mice treated with poly IC at -1 day (-1D) or +8 days (+8D) or untreated (N) were determined at 14 dpi using quantitative PCR. Data were expressed by fold induction after normalization to the GAPDH mRNA levels. The values given are means ± standard deviation of triplicate. Statistical significances of the differences were indicated with asterisks (*, *P *< 0.05; **, *P *< 0.01; ***, *P *< 0.001). Click here for file
