# Supplementary figures and images for: TLR3 signaling is either protective or pathogenic for the development of Theiler's virus-induced demyelinating disease depending on the time of viral infection
Source: J Neuroinflammation. 2011 Dec 21;8:178. doi: 10.1186/1742-2094-8-178 (PMC3293102; doi:10.1186/1742-2094-8-178)

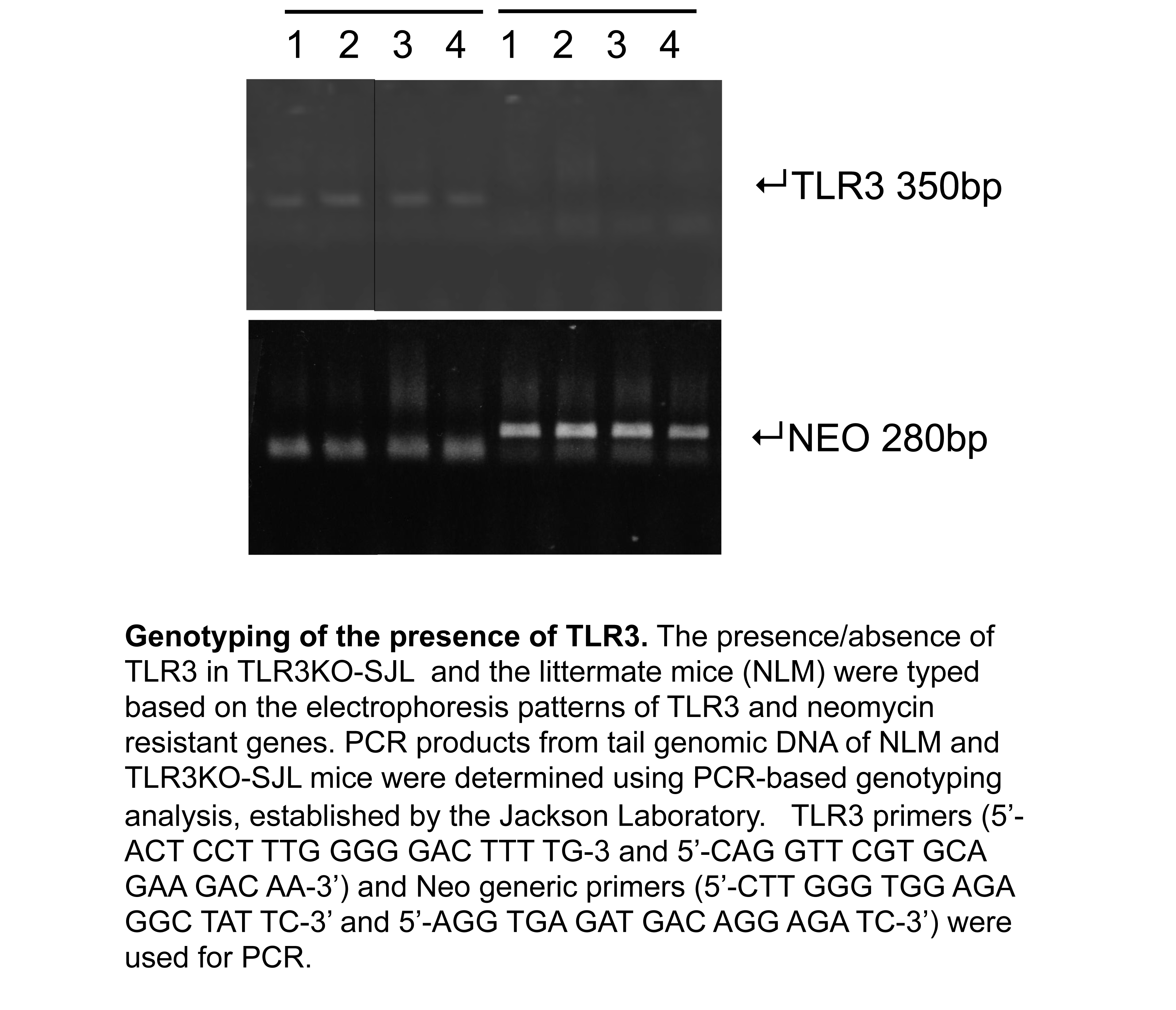

Supplement: Additional file 1 — Genotyping of the presence of TLR3. The presence/absence of TLR3 in TLR3KO-SJL and the littermate mice (NLM) were typed based on the electrophoresis patterns of TLR3 and neomycin resistant genes. PCR products from tail genomic DNA of NLM and TLR£KO-SJL mice were determined using PCR-based genotyping analysis, established by the Jackson Laboratory. TLR3 primers (5'-ACT CCT TTG GGG GAC TTT TG-3 and 5'-CAG GTT CGT GCA GAA GAC AA-3') and Neo generic primers (5'-CTT GGG TGG AGA GGC TAT TC-3' and 5'-AGG TGA GAT GAC AGG AGA TC-3') were used for PCR. [file 1742-2094-8-178-S1.TIFF]

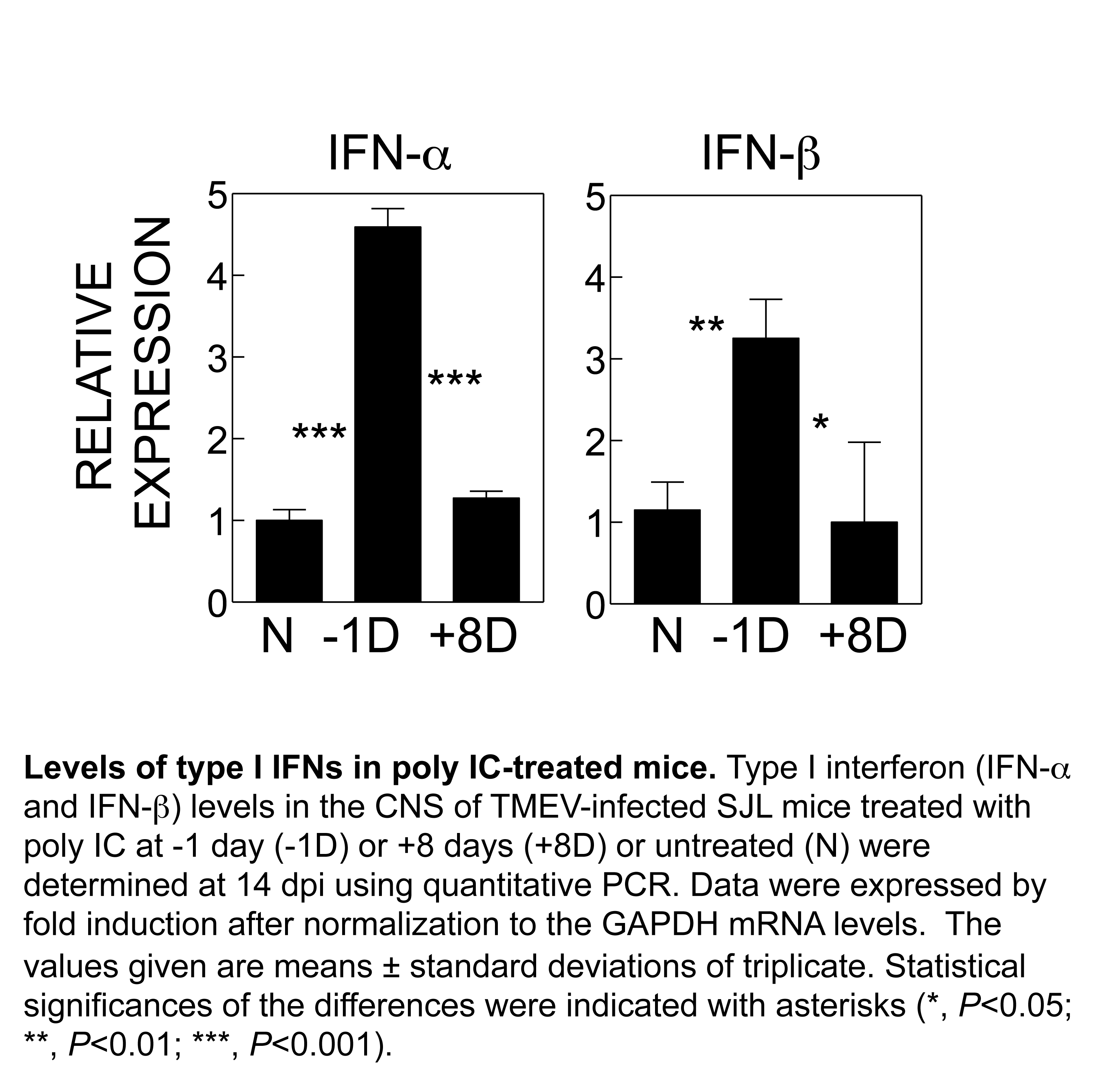

Supplement: Additional file 2 — Levels of type I IFNs in poly IC-treated mice. Type I interferon (IFN-α and IFN-β) levels in the CNS of TMEV-infected SJL mice treated with poly IC at -1 day (-1D) or +8 days (+8D) or untreated (N) were determined at 14 dpi using quantitative PCR. Data were expressed by fold induction after normalization to the GAPDH mRNA levels. The values given are means ± standard deviation of triplicate. Statistical significances of the differences were indicated with asterisks (*, P < 0.05; **, P < 0.01; ***, P < 0.001). [file 1742-2094-8-178-S2.TIFF]
